# Interruptions in treatment among adults on anti-retroviral therapy before and after test-and-treat policy in Tanzania

**DOI:** 10.1371/journal.pone.0292740

**Published:** 2023-11-15

**Authors:** Redempta J. Mbatia, Expeditho L. Mtisi, Abbas Ismail, Christopher V. Henjewele, Sisty J. Moshi, Alexander K. Christopher, Noela W. Nsanzugwanko, Appolinary G. Bukuku, Rehema A. Msimbe, Agnes R. Kirato, Francis S. Nyabukene, Eunice J. Mmari, Anath A. Rwebembera, Benedicta N. Masanja, Alexander Kailembo, Eva J. Matiko

**Affiliations:** 1 Tanzania Health Promotion Support (THPS), Dar es Salaam, Tanzania; 2 Department of General Studies, Dar es Salaam Institute of Technology, Dar es Salaam, Tanzania; 3 US Center for Disease Control and Prevention (CDC), Tanzania Country Office, Dar es Salaam, Tanzania; 4 Ministry of Health, Community Development, Gender, Elderly and Children, Dodoma, Tanzania; Institut Pasteur Cambodia: Institut Pasteur du Cambodge, CAMBODIA

## Abstract

**Introduction:**

The World Health Organization recommended the initiation of antiretroviral therapy (ART) for people living with HIV (PLHIV) regardless of CD4 cell counts. Tanzania adopted this recommendation known as test-and-treat policy in 2016. However, programmatic implementation of this policy has not been assessed since its initiation. The objective of the study was to assess the impact of this policy in Tanzania.

**Methods:**

This was a cross-sectional study among PLHIV aged 15 years and older using routinely collected program data. The dependent variable was interruption in treatment (IIT), defined as no clinical contact for at least 90 days after the last clinical appointment. The main independent variable was test-and-treat policy status which categorized PLHIV into the before and after groups. Co-variates were age, sex, facility type, clinical stage, CD4 count, ART duration, and body mass index. The associations were assessed using the generalized estimating equation with inverse probability weighting.

**Results:**

The study involved 33,979 PLHIV—14,442 (42.5%) and 19,537 (57.5%) were in the before and after the policy groups, respectively. Among those who experienced IIT, 4,219 (29%) and 7,322 (38%) were in the before and after the policy groups respectively. Multivariable analysis showed PLHIV after the policy was instated had twice [AOR 2.03; 95%CI 1.74–2.38] the odds of experiencing IIT than those before the policy was adopted. Additionally, higher odds of experiencing IIT were observed among younger adults, males, and those with advanced HIV disease.

**Conclusion:**

Demographic and clinical status variables were associated with IIT, as well as the test-and-treat policy. To achieve epidemic control, programmatic adjustments on continuity of treatment may are needed to complement the programmatic implementation of the policy.

## Introduction

There are an estimated 38 million people living with HIV (PLHIV) globally as per the Joint United Nations programme on HIV/AIDS (UNAIDS) report, with 1.5 million newly diagnosed with HIV and about 700,000 people died from AIDS-related illnesses in 2020 [1]. With two-thirds of PLHIV coming from sub-Saharan Africa (SSA), the region bears the largest burden of this global epidemic including new HIV infections and AIDS-related deaths. Furthermore, Eastern and Southern African countries make for half of the 38 million PLHIV [1].

Tanzania, an Eastern African country with a population of 63 million people [2], has not been spared from the devastating impact of the HIV epidemic. According to the 2016–2017 Tanzania HIV Impact Survey (THIS), HIV prevalence among people aged 15 years and older was 4.9%, which corresponded to approximately 1.4 million PLHIV and new cases of HIV infection were estimated at 72,000 [3]. Additionally, a 2020 UNAIDS report estimated 68,000 new HIV infections and 32,000 AIDS-related deaths in Tanzania [4]. With the sheer number of new HIV infections, the attainment of the global 95-95-95 UNAIDS targets will rely on achievement of early identification and initiation of antiretroviral therapy (ART), improved continuity of treatment and virological success of PLHIV.

The World Health Organization (WHO) provided recommendations in 2016 to initiate ART to all PLHIV regardless of WHO clinical stage and at any CD4 cell count—a test-and-treat policy. This was an update to the 2013 recommendations to initiate ART for all adults with HIV and a CD4 count at or below 500 cells/mm3 [5, 6]. Furthermore, WHO published additional guidelines in 2017 to recommend rapid ART initiation in the era of test-and-treat policy which defined rapid initiation as ART initiation within seven days from the day of HIV diagnosis [7]. The latest WHO recommendations published in 2021 maintained the 2017 recommendations of rapid ART initiation [8].

Benefits of rapid ART initiation as part of test-and-treat policy have been widely reported in the literature. The primary objective of ART is to prevent HIV-related morbidity and mortality and to reduce the risk of HIV transmission to others [9]. Moreover, studies have reported on timing of ART initiation whereby rapid ART initiation has been associated with several advantages such as increased ART uptake, better linkage and engagement to care, improved continuity of treatment, virologic suppression achievement and reduced mortality. In various studies, diverse regions have reported positive outcomes associated with different approaches to initiating ART. For instance, a study conducted in South Africa highlighted a greater likelihood of achieving viral suppression among participants who received immediate ART [10]. Similarly, a study in Haiti revealed improved retention in care, specifically among participants who were provided with ART on the same day as testing [11]. In yet another study conducted in Lesotho, there was a notable increase in linkage to care among participants who were offered same-day ART [12]. Studies in high-income countries have also reported similar favorable outcomes to rapid ART initiation including demonstration of safety and acceptability of same-day ART initiation [13], and shortening the time to virologic suppression [14]. With clear evidence and WHO recommendations on test-and-treat guidelines, more than 130 countries globally have scaled-up ART uptake and most of the low-and middle-income countries including Tanzania have adopted the test-and-treat policy [15].

Tanzania adopted the test-and-treat recommendations in October 2016. However, since the adoption of this policy in the country, information on its programmatic impact is limited. A local non-governmental organization (NGO), Tanzania Health Promotion Support (THPS), completed a five-year project from 2013 to 2018 with funding from the U.S. President’s Emergency Plan for AIDS Relief (PEPFAR) through the US Centers for Disease Control and Prevention (CDC). THPS supported the government of Tanzania to scale up HIV services in the regions of Kigoma, Pwani and Zanzibar. This study was designed to assess the impact of the test-and-treat policy implemented by THPS among PLHIV before and after the initiation of the policy in Tanzania.

## Methods

### Data source

This study used national program data obtained from the care and treatment clinic (CTC2) database which are under the custodianship of the National AIDS Control Program (NACP) of the Ministry of Health. The CTC2 database is based on the national HIV care and treatment monitoring tools which capture client level data of all ages for HIV services rendered and clinical outcomes including demographic information, testing services, type and duration of ARVs dispensed, and the clinical status of the patients. Data extracted from the CTC2 database were first de-identified to omit personally identifiable information before being analyzed for the study.

### Study design and data collection

This was a cross-sectional study using CTC2 data from October 2014 to September 2018. During clinic visits, clients are seen by health care workers who collect information through face-to-face interviews and clinical examinations. The collected data are recorded on the paper-based CTC registers. Thereafter, data from the CTC registers are entered in the CTC2 database by data officers at the sites. All data were fully anonymized using participant and site ID numbers unlinked to any personal identifiers. This analysis was done following ethical approval by Tanzania National Institute for Medical Research (NIMR) and Center for Global Health Associate Director for Science, Atlanta, GA. A waiver of informed consent was granted for this analysis.

### Study sample and population

The study included 33,979 PLHIV who were initiated on ART across 126 THPS supported facilities from Pwani, Kigoma and Zanzibar regions. Out of those, 14,442 were initiated on ART between October 2014 and September 2016 corresponding the period before implementation of the test-and-treat policy—the “before” group. And 19,537 were initiated ART between October 2016 and September 2018 corresponding the period after implementation of the test-and-treat policy—the “after” group.

### Study variables

The dependent variable was a binary variable measuring interruption in treatment (IIT). IIT was defined as PLHIV with no clinical contact for at least 90 days after the last clinical appointment. Those who either never interrupted treatment or had clinical contact within 90 days after the last clinical appointment were categorized to have not experienced IIT. The definition of IIT has varied over time. For instance, the current PEPFAR definition considers IIT as no clinical contact for at least 28 days after the last clinical appointment. We used the older definition because it was the one utilized at the time of the study.

The main exposure of interest was test-and-treat policy status which categorized PLHIV into the before and after groups. Co-variates included in the study were age groups (15–24 years, 25–34 years, 35–44 years, 45 years and older), sex (male vs. female), region (Kigoma vs. Pwani vs. Zanzibar), facility type (hospital vs. health center vs. dispensary), WHO clinical stages (1-IV), CD4 cell count groups (<350 vs. 350–500 vs. >500), and duration on ART (< = 1 year vs. >1 year) and body mass index (<18.5 vs. 18.5-<25 vs. 25-<30 vs. > = 30).

### Statistical analysis

Absolute numbers and percentages were used to describe demographic and clinical characteristics for PLHIV initiated on ART before and after implementation of the test-and-treat policy. Additionally, descriptive statistics reporting absolute numbers and percentages were reported for PLHIV who experienced IIT before and after implementation of the policy by demographic and clinical characteristics. Univariable and multivariable generalized estimating equation models with inverse probability weighting were used to determine the associations between the independent variables and IIT. The independent variables with p-value <0.2 in the univariable models were considered for multivariable analysis. Odds ratios (OR) and 95% confidence intervals (95%CI) were reported. Statistical analyses were performed using the SAS ^®^ statistical software package, Release 9.4 (Cary, North Carolina, USA).

## Results

Table 1 describes the demographic and clinical characteristics of the study population. The majority of PLHIV were aged 25–44 years (62%), were female (69%), and were from Pwani region (65%). Additionally, higher proportions of PLHIV were registered from health centers (42.1%), had WHO clinical stage I (45%), and had CD4 cell count <350 (49%). Overall, around one-third (34%) of the study population experienced IIT. Descriptive comparison of PLHIV in the before and after test-and-treat policy groups showed higher proportion of IIT among PLHIV in the after group (38%) compared to those in the before group (29%). In addition, the proportions of individuals with WHO clinical stage I and CD4 count >500 were higher among PLHIV after the policy (WHO stage I: 51% in the after group vs. 38% in the before group and CD4 count: 37% in the after group vs. 25% in the before group).

**Table 1:** Demographic and clinical characteristics of the study population by test-and- start policy status.

Characteristic	All Clients (N = 33,979)	Before Policy (N = 14,442)	After Policy (N = 19,537)	P-values
	n (%)	n (%)	n (%)	
**Age (years)**				0.0002
Median (IQR)	38(16)	38(15)	38(16)	
15–24	3,975 (11.7)	1,581 (11.0)	2,394 (12.3)	
25–34	10,528 (31.0)	4,455 (30.9)	6,073 (31.1)	
35–44	10,434 (30.7)	4,572 (31.7)	5,862 (30.0)	
45+	9008 (26.5)	3,813 (26.4)	5,195 (26.6)	
**Sex**				< .0001
Male	10,536 (31.0)	4,124 (28.6)	6,412 (32.8)	
**Region**				< .0001
Kigoma	9,240 (27.3)	3,650 (25.4)	5,592 (28.7)	
Pwani	22,051 (65.1)	9,631 (66.9)	12,420 (63.7)	
Zanzibar	2,584 (7.6)	1,109 (7.7)	1,475 (7.6)	
**Facility Type**				< .0001
Hospital	11,613(34.3)	4,894(34.0)	6,719(34.5)	
Health center	14,287(42.1)	6,259(43.4)	8,028(41.2)	
Dispensary	7,992(23.6)	3,252(22.6)	4,740(24.3)	
**WHO Clinical stage**				< .0001
I	15,252 (45.3)	5,381 (37.7)	9,871 (51.0)	
II	10,859 (32.3)	4,865 (34.0)	5,994 (31.0)	
III	6,138 (18.3)	3,346 (23.4)	2,792 (14.4)	
IV	1,391 (4.1)	694 (4.9)	697 (3.6)	
**CD4 Cell Count (cells/mm3)**				< .0001
Median (IQR)	430(361)	387(333)	516(380)	
< 350	6,330 (49.4)	4,061 (54.6)	2,269 (42.1)	
350–500	2,647 (20.6)	1,533 (20.6)	1,114 (20.7)	
> 500	3,850 (30.0)	1,845 (24.8)	2,005 (37.2)	
**ART Duration**				< .0001
Median (IQR) (years)	1(1)	1(1)	2(1)	
< 1 year	18,421(72.7)	7,428(70.4)	10,993(74.3)	
> = 1 year	6,918(27.3)	3,115(29.6)	3,803(25.7)	
**Body Mass Index (kg/m** ^**2**^ **)**				< .0001
Median (IQR)	22(6)	21(5)	22(6)	
<18.5	4,550(13.4)	1,818(12.6)	2,732(14.0)	
18.5-<25	24,569(72.3)	11,015(76.3)	13,554(69.4)	
25-<30	3,257 (9.6)	1,078(7.5)	2,179(11.1)	
30+	1,588(4.7)	516(3.6)	1,072(5.5)	
**Interruption in Treatment**				< .0001
Yes	11,553(34.0)	4,219(29.2)	7,322(37.5)	

Table 2 describes IIT by demographic and clinical characteristics of the study population in the before and after groups of the test-and-treat policy. The age was significantly associated with IIT in both the before and after groups with young adults having the highest proportion of IIT (45% in the after group and 38% in the before group). Around two-third of PLHIV who experienced IIT were females, in both before policy (71%) and after policy (67%) groups. Higher proportions of IIT were observed in both groups among PLHIV with WHO stages III and IV (WHO stage IV: 51% in the after group and 46% in the before group). Likewise, higher proportions of IIT were also observed in both groups among PLHIV with CD4 cell count <350 (CD4 cell count <350: 40% in the after group and 39% in the before group). Furthermore, PLHIV who were on ART for less than a year had higher proportions of IIT in both groups (55% in the after group and 45% in the before group).

**Table 2:** Number of PLHIV who experienced interruption in treatment across study characteristics by test-and-start policy status.

Characteristic	IIT Before Policy (N = 4,219/14,442)	P-values	IIT After Policy (N = 7,322/19,537)	P-values
	n (%)		n (%)	
**Age (years)**		< .0001		< .0001
15–24	607 (38.2)		1,130 (45.3)	
25–34	1,400 (32.1)		2,514 (41.3)	
35–44	1,214 (26.5)		1,979 (35.0)	
45+	996 (26.1)		1,686 (32.5)	
**Sex**		< .0001		0.006
Male	1,315 (31.8)		2,492 (38.9)	
**Region**		0.01		< .0001
Kigoma	1,016 (27.8)		2,104 (37.6)	
Pwani	2,833 (29.4)		4,792 (38.6)	
Zanzibar	361 (32.6)		396 (26.8)	
**Facility Type**		0.01		0.52
Hospital	1,371(28.0)		2,502(37.2)	
Health center	1,825(29.2)		3,040(37.9)	
Dispensary	1,014(31.2)		1,750(36.9)	
**WHO Clinical stage**		< .0001		< .0001
I	1,539(28.6)		3,622(36.7)	
II	1,210(24.8)		2,082(34.7)	
III	1,107(33.0)		1,204(43.1)	
IV	320(46.2)		556(51.1)	
**CD4 Cell Count(cells/mm3)**		< .0001		0.003
< 350	1,586 (39.0)		916 (40.4)	
350–500	331(21.6)		406 (36.4)	
> 500	355 (19.2)		713 (35.5)	
**ART Duration**		< .0001		< .0001
< 1 year	3,338(44.9)		6,001(54.6)	
> = 1 year	591(19.0)		971(25.5)	
**Body Mass Index(kg/m** ^**2**^ **)**		0.001		0.05
<18.5	565(31.0)		1,050(38.4)	
18.5-<25	3,251(29.5)		5,117(37.8)	
25-<30	279(25.9)		786(36.1)	
30+	124(24.0)		369(34.4)	

Table 3 describes factors associated with IIT, including test-and-treat policy as our exposure of interest. In the multivariable model, PLHIV in the after test-and-treat group had twice the odds [AOR 2.03; 95%CI 1.74–2.38] of experiencing IIT compared to those in the before group. Additionally, the model showed that younger adults (15–24 years) have significantly higher odds of experiencing IIT [AOR 1.52; 95%CI 1.31–1.75]. Males had 14% higher odds of IIT [AOR 1.14; 95%CI 1.01–1.28] compared to females. PLHIV in WHO stage IV had the highest odds of experiencing IIT [AOR 1.16; 95CI 1.10–1.22] compared with those in WHO stage I, when all other variables remained constant. Likewise, PLHIV with CD4 cell counts <350 cells/mm^3^ had the highest odds of experiencing IIT [AOR 1.13; 95%CI 1.11–1.16] compared with those with CD4 > 500 cells/mm^3^.

**Table 3:** Univariable and multivariable associations of interruption in treatment by the study characteristics.

Characteristic	Univariable Model		Multivariable Model	
	OR (95% CI)	P-values	AOR (95% CI)	P-values
**Age (years)**		< .0001		< .0001
15–24	1.63(1.44–1.85)		1.52(1.31–1.75)	
25–34	1.43(1.29–1.58)		1.44(1.28–1.62)	
35–44	1.02(0.91–1.15)		1.07(0.93–1.22)	
45+	Reference		Reference	
**Sex**		.0012		0.02
Female	Reference		Reference	
Male	1.15 (1.05–1.26)		1.14 (1.01–1.28)	
**Region**		0.17		0.65
Kigoma	0.99(0.88–1.11)		0.95(0.83–1.08)	
Pwani	0.93(0.83–1.04)		0.94(0.83–1.07)	
Zanzibar	Reference		Reference	
**Facility Type**		0.48		
Hospital	0.92(0.80–1.06)			
Health center	0.91(0.77–1.08)			
Dispensary	Reference			
**WHO Clinical stage**		< .0001		< .0001
I	Reference		Reference	
II	0.97(0.95–1.00)		1.02(0.99–1.04)	
III	1.03(1.00–1.06)		1.08(1.05–1.12)	
IV	1.15(1.10–1.21)		1.16(1.10–1.22)	
**CD4 Cell Count(cells/mm3)**		< .0001		< .0001
< 350	1.16(1.14–1.18)		1.13(1.11–1.16)	
350–500	1.02(1.01–1.04)		0.99(0.98–1.01)	
> 500	Reference		Reference	
**ART Duration**		< .0001		< .0001
< 1 year	3.55(2.75–4.58)		3.50(2.84–4.33)	
> = 1 year	Reference		Reference	
**Body Mass Index(kg/m** ^**2**^ **)**		< .0001		0.0003
<18.5	1.04(1.02–1.05)		1.02(1.00–1.03)	
18.5-<25	Reference		Reference	
25-<30	0.98(0.97–0.99)		0.97(0.95–0.98)	
30+	0.98(0.96–1.01)		0.95(0.92–0.99)	
**Policy**		< .0001		< .0001
Before policy	Reference		Reference	
After policy	1.47(1.32–1.63)		2.03(1.74–2.38)	

## Discussion

In this study, the majority of PLHIV who experienced IIT were initiated on treatment after the test-and-treat policy. Evidence in the literature for this finding is mixed. There are studies which have shown improved continuity of treatment among PLHIV who rapidly initiated ART [11, 16], and there are other studies which have reported the opposite. For example, studies in France and the United States reported that rapid ART initiation did not seem to improve continuity of treatment [17, 18], while studies in sub-Saharan Africa have reported that PLHIV who rapidly initiate ART experienced greater IIT [19, 20]. Some of the reasons reported in these studies include PLHIV’s lack of preparedness, education and motivation for HIV treatment, attitudes of healthcare workers, inflexible clinic operations, and lack of nearby ART sites.

For the context of Tanzania, the test-and-treat era meant provision of 14-day supply of ART on the same-day or within seven days of HIV diagnosis. This change of rapid ART initiation may have had a negative effect on treatment adherence due to the inability to adequately prepare PLHIV for ART initiation and address barriers such as disbelief, acceptance and stigma [21]. Before the implementation of the test-and-treat policy, the guidelines in Tanzania required PLHIV to initiate ART after an average of three adherence sessions conducted by health care providers. These multiple visits provided an opportunity for PLHIV to have greater client-provider interactions to address concerns and offer support. This opportunity is missed in the test-and-treat era and may have contributed to the observed higher rates of IIT. However, it is important to be cautious in the interpretation of these findings since our IIT definition is limited and does not capture other measures of treatment adherence such adherence to taking medications and longer-term adherence.

The study findings also showed that advanced WHO clinical stages and low CD4 counts were associated with higher IIT. Effects of IIT have been widely reported in the literature and linked with viral non-suppression, opportunistic infections, increased risk of transmission, and increased morbidity and mortality [22, 23]. To improve options and access to care for PLHIV in the test-and-treat era, the government of Tanzania worked with stakeholders to implement differentiated service delivery (DSD) models. These models differ from the conventional model in terms of location and frequency of contacts with the health facilities, cadre of provider involved, and/or types of services provided. These include community ART services, multi-month ARV dispensing, optimization of ART, linkage case management, treatment support groups, and friendly working hours [24].

In the context of Tanzania, many of these DSD interventions had been established at the beginning of the test-and-treat era, but full scale up to all regions in the country took time because implementation was completed in phases. This means that at the time of data collection for this study, many of these interventions were not actively implemented in the THPS regions. For instance, community ART outreach services and linkage case management from the Bukoba Combination Prevention Evaluation (BCPE) models [25, 26], had not been rolled out to all supported facilities across THPS regions. This delay of universal implementation of DSD models in the era of test-and-treat policy may have attributed to the reported higher IIT, but a direct association cannot be established since our analyses did not measure DSD interventions.

Although our study highlights important findings during the initial years of the implementation of the test-and-treat policy, there is a need to re-assess the impact of the policy in the current times. It may be hypothesized that rapid ART initiation coupled with scaled-up implementation of DSD strategies such as extended linkage case management, expanded community outreach services, multi-month dispensing, and teen/men ART refill clubs might have a positive impact on continuity of treatment especially to the identified high risk sub-populations such as young adults, men, and those with advanced HIV diseases. Identified factors associated with treatment interruption in this study may be addressed through integration of DSD models with the test-and-treat policy leading to advancement towards reaching the UNAIDS 95-95-95 goals by 2030.

This is the first study in Tanzania to assess the impact of the test-and-treat policy on IIT at a large scale using programmatic routine data. THPS had a unique advantage of being the implementing partner in the regions of Kigoma, Pwani and Zanzibar before and after the initiation of the policy. However, the study has limitations. Various clinical outcome variables such as viral load suppression, kidney and liver function tests were not used due to missingness or lack of data. For routine data, issues related to missing data including CD4 counts are common. Additionally, viral load suppression, an important outcome of successful ART usage, was not a standard of care before the initiation of test-and-treat policy. Other study limitations include lack of assessment of social and cultural factors affecting PLHIV on ART due to lack of inclusion of those aspects in the data source.

## Conclusion

In this study, PLHIV who were initiated on ART after the test-and treat policy were more likely to experience IIT. To improve outcomes in the era of test-and-treat, programmatic adjustments which improve continuity of treatment for PLHIV may be needed to complement the implementation of the policy. Additionally, further research could help to better understand the current impact of the test-and-treat policy after more than five years of implementation.
